# Role of Bone Marrow-Derived Monocytes/Macrophages in the Repair of Mucosal Damage Caused by Irradiation and/or Anticancer Drugs in Colitis Model

**DOI:** 10.1155/2010/634145

**Published:** 2011-01-04

**Authors:** Junji Takaba, Yuji Mishima, Kiyohiko Hatake, Tadashi Kasahara

**Affiliations:** ^1^Department of Biochemistry, Graduate School of Pharmaceutical Sciences, Keio University, 1-5-30, Shibakoen, Minato-ku, Tokyo 105-8512, Japan; ^2^Department of Clinical Research, Cancer Chemotherapy Center, Japanese Foundation for Cancer Research, 3-10-6 Ariake, Koto-ku, Tokyo 135-0063, Japan

## Abstract

Mucosal damage is a common side effect of many cancer treatments, especially radiotherapy and intensive chemotherapy, which often induce bone marrow (BM) suppression. We observed that acetic acid- (AA-) induced mucosal damage in the colon of mice was worsened by simultaneous treatment with irradiation or 5-FU. However, irradiation 14 days prior to the AA treatment augmented the recovery from mucosal damage, suggesting that the recovery from BM suppression had an advantageous effect on the mucosal repair. In addition, BM transplantation also augmented the recovery from AA-induced mucosal damage. We further confirmed that transplanted BM-derived cells, particularly F4/80^+^Gr1^+^ “inflammatory” monocytes (Subset 1), accumulated in the damaged mucosal area in the early healing phase, and both of Subset 1 and F4/80^+^Gr1^−^ “resident” monocytes (Subset 2) accumulated in this area in later phases. Our results suggest that monocytes/macrophages contribute to the mucosal recovery and regeneration following mucosal damage by anticancer drug therapy.

## 1. Introduction

Mucosal damage is a common side effect of many cancer treatments, especially intensive chemotherapy and radiotherapy. This mucosal damage can seriously affect the quality of life of patients seriously and may sometimes demand changes in or limit the therapy [1–3]. Intestinal mucositis and oral mucositis are significant clinical problems in patients receiving ionizing radiation as the intestinal and oral mucosae are the most radiosensitive organs. Chemotherapy-induced intestinal mucositis involves multiple mechanisms, including induction of crypt cell apoptosis and cytostasis, which implies the possible involvement of pathways relating to p53, the Bcl-2 family, and caspases [3–5]. Further, the nuclear factor-kappa B [6], the cyclooxygenase pathway [7], and epigenetic aberrations [8] have been implicated in the regulation of radiation-induced mucositis. Thus, these findings have lead to further research into the protection and rescue of the intestinal mucosal damages in using the experimental animals. Namely, lysophosphatidic acid [9], *Ginkgo biloba* extract [10], and D-methionine [11] have been shown to exhibit protective action against radiation-induced organ damage *in vitro* and *in vivo*. Recently, histone deacetylase inhibitor [8], lovastatin [12], and ascorbic acid [13] have also been shown to protect against radiation-induced mucositis. Proinflammatory cytokines have also been implicated in the pathophysiology of chemotherapy-induced gastrointestinal mucositis [14]. However, whether these cytokines play a key role in the development and healing of radiotherapy-induced gastrointestinal mucositis remains unknown. 

 In the present study, we employed an acetic acid- (AA-) induced mucosal damage animal model, an established model for the screening of the anti-inflammatory drugs [15, 16], to examine the effects of irradiation or 5-FU on the healing process following AA-induced mucosal damage. Surprisingly, we found that irradiation prior to AA-induced mucosal damage enhanced mucosal healing, while the simultaneous irradiation or 5-FU treatment simultaneously with AA treatment exacerbated the mucosal damage. Since irradiation or anticancer drugs therapy also induces severe leukocytopenia, the effect of bone marrow transplantation (BMT) on mucosal healing was also studied. In particular, we studied the two monocyte/macrophage subsets, “inflammatory” and “resident” [17–21], to determine how these monocyte/macrophage subsets migrate to the mucosal damaged lesion and to elucidate their roles in mucosal healing. 

 Several reports have shown that monocytes/macrophages contribute to the healing of wounds arising from vascular injury [22], spinal cord injury [23], and myocardial infarction [24]. Still other studies have indicated that monocytes/macrophages may promote wound healing following skin injury [25–28]. However, the role of monocyte/macrophage subsets in the gastrointestinal mucositis has not been elucidated. Here, we propose a role of monocytes/macrophages in the protection against the intestinal mucosal damage.

## 2. Materials and Methods

### 2.1. Mice and Total Body Irradiation (TBI)

C57BL/6J male mice, aged 6 weeks, were purchased from Charles River Japan, Inc. (Yokohama). The mice were maintained under specific pathogen-free conditions and provided with sterile food and water *ad libitum*. The animals were handled and treated in accordance with the guidelines set forth by the animal use and care committee of the Japanese Foundation for Cancer Research, and all procedures conformed to the NIH guide for the *Care and Use of Laboratory Animals*. The mice were irradiated totally with a total dose of 4 Gy per animal using an X-ray generator as a radiation therapy and designated as RT mice. The irradiated mice were bred in a filter-topped cage to prevent infection. The absolute count of white blood cells (WBCs) was analyzed using an F-820 semi-automated hematology analyzer (Sysmex, Kobe, Japan).

### 2.2. Induction of Experimental Mucosal Damage

Twenty microliters of 3% AA (Wako, Tokyo, Japan) was injected into the lumen of the colon and retained for 20 s using round-ended tweezers. Thereafter, the lumen was washed with saline and stitched closed as described previously [9, 15, 16]. Prior to or after mucosal damage, mice were administered 5-FU (100 mg/kg) (AA + 5-FU) i.v. via tail vein or radiation (4 Gy) (AA + Radiation Therapy (RT)) as indicated. On the day of evaluation, the colon was longitudinally incised and spread open, and the size of the damaged area (length × width (mm^2^)) was determined using a stereoscopic microscope.

### 2.3. BMT and Cell Labeling

BMCs were flushed from the femur and tibia with RPMI 1640 medium using a 26 G 1/2 needle. To remove debris, the suspension was filtered through a 35 *μ*m cell strainer (Falcon). The cells were then washed with medium and counted, and 1 × 10^7^ cells were injected into the tail vein of each of the mucosally damaged mice. To enable tracking analysis, BMCs were stained with VivoTag 750 (VT750; Ex: 750 ± 5 nm, Em: 775 ± 5 nm, MW: 980 Da) to visualize the accumulation of cells in vivo. VT750 was commercially obtained from VisEn Medical (Woburn, MA; now PerkinElmer Heath Sciences, Inc.). Cells were incubated with 30 *μ*g/ml VT750 for 30 min at 37°C and washed twice after labeling. For further analysis, BMCs were stained with 10 *μ*M CFDA-SE (Carboxyfluorescein diacetate, succinimidyl ester; Molecular probes; Invitrogen) for 15 min at 37°C. The cells were then repelleted by centrifugation, resuspended in fresh, prewarmed medium, and incubated for 30 min at room temperature to ensure complete incorporation of the probe before a final washing step.

### 2.4. Preparation of Damaged Mucosa and Identification of Injected BMCs

Dissected mucosa was incubated in calcium and magnesium-free HBSS (GIBCO) containing 2.5% heat-inactivated FBS and 1 mM DTT (Sigma-Aldrich) to remove mucus. The mucosa was then incubated in HBSS containing 1 mM EDTA (GIBCO) for 45 min at 37°C. Tissues were then collected and incubated in HBSS containing 1 mg/ml collagenase (Nacalai Tesque, Tokyo, Japan) and 0.1 mg/ml DNase I for 60 min at 37°C, and cells were washed and reacted with an antibody for flow cytometric analysis.

### 2.5. Flow Cytometric Analysis

The following monoclonal antibodies and staining reagents were used in combination according to the manufacturers' protocols: Phycoerythrin-(PE-) conjugated CD11b (BD Pharmingen), Gr1 (Ly6C/G; BD Pharmingen), Peridinin Chlorophyll Protein-Cyanin 5.5-(PerCP-Cy5.5-) conjugated anti-CD11b (BD Pharmingen), and allophycocyanin- (APC-) conjugated anti-CD62L (Beckman Coulter), anti-Gr1 (Ly6C/G) (BD Pharmingen), or anti-F4/80 (Caltag). To exclude nonviable cells, 7-amino-actinomycin D (7-AAD) was used. Cells were stored in cold conditions and protected from light; they were analyzed approximately 15 min after addition of 7-AAD. Thereafter, cells were analyzed on an FACSCalibur flow cytometer (Becton Dickinson, Franklin Lakes, NJ) using CellQuest software (Becton Dickinson).

### 2.6. Confocal Microscopic Analysis

*In vivo* monitoring of cell migration was performed using the AA + 5-FU model. On day 0, the mice were treated with AA and 5-FU (100 mg/kg), and after 24 h, 1 × 10^7^ VT750-labeled BMCs were injected into the mice via the tail vein. On day 2, the colon was excised from the mice, and the damaged area of the mucosa was longitudinally incised and spread out for laser scanning microscopy (Olympus IV100).

### 2.7. Statistical Analysis

Data are expressed as mean ± SE. Data were analyzed using Welch's *t*-test to compare between two groups, Tukey's method was used between three groups, and differences with *P* < .05 were considered statistically significant.

## 3. Results

### 3.1. TBI-Induced Leukocytopenia and Thrombocytopenia

**Figure 1:**
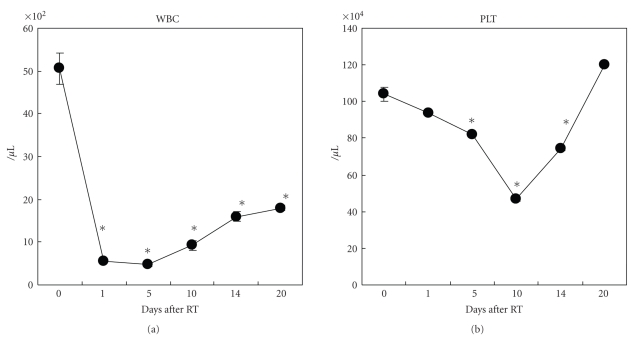
Total body irradiation (TBI) as a radiation therapy (RT) induced leukocytopenia and thrombocytopenia. Twenty-five mice were divided into 5 groups, and each group was irradiated by a dose of 4 Gy on day 0. Mice were sacrificed, and white blood cells (WBC) were counted using a semiautomated hematology analyzer F-820 (Sysmex) on day 1, 5, 10, 14, and 20, respectively (*n* = 5/each time point). (a) WBC number decreased after TBI, and the low value continued around day 5. (b) Platelets number also decreased, and the lowest number was observed around day 10. Values represent mean ± SEM. *P < .05 versus day 0. Data were analyzed using the Tukey method.

It is well known that TBI induces mucosal injury to cancer patients. In order to mimic similar condition, we chose TBI as a radiotherapy (RT) but not local irradiation. A dose of 4 Gy TBI induced leukocytopenia and thrombocytopenia in mice (Figures 1(a), 1(b)). TBI drastically reduced the WBC count within 1 day; the WBC nadir continued for 1 week and then gradually tended to recover. Similarly, the platelet count decreased; however, the decrease was less severe, with the nadir occurring on day 10. We therefore assumed that day 14 after TBI represented a favorable stage in the recovery phase for both WBCs and platelets.

### 3.2. The Relationship between Mucosal Damage and Irradiation

**Figure 2:**
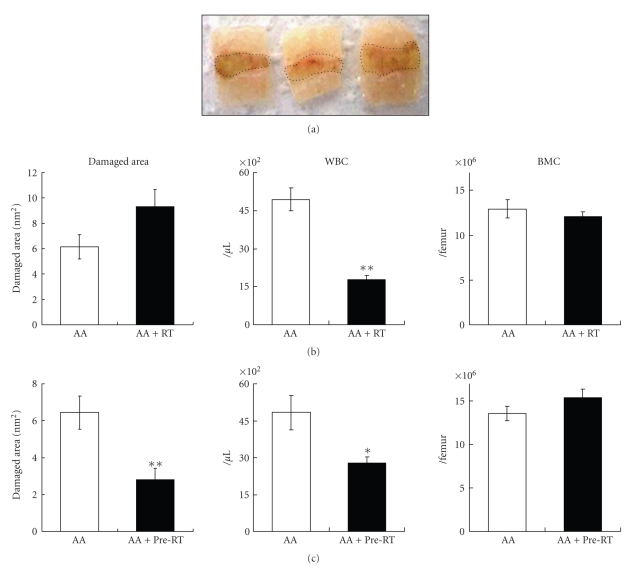
Effect of pre- or simultaneous irradiation on the acetic acid (AA-) induced mucosal damage. (a) Mucosal damage in the colon induced by 3% AA. On the evaluated day, colon was longitudinally incised and spread. Damaged area was evaluated with stereoscopic microscope. (b) Mice were induced mucosal damage on day 0 (AA) and irradiated on the same day (AA + RT). WBC and bone marrow cells (BMC) on day 7 were indicated in the right panels. (c) Mice were AA treated (AA) or irradiated 14 days before AA treatment (AA + Pre-RT). Note that the damaged area (AA + Pre-RT) was smaller than nonirradiated control (AA), indicating that preirradiation enhanced healing of mucosal damage. WBC and BMC on day 7 were indicated in the right panels. Values represent mean ± SEM. *P < .05, **P < .01. Data were analyzed using Welch's *t*-test.

Experimental mucosal damage in the colon was induced by 3% AA. To evaluate mucosal damage, the colon was removed, longitudinally incised, and spread out. The area of the damaged zone was then measured using a stereoscopic microscope (Figure 2(a)). Concomitantly, we examined the effect of RT on mucosal damage in the colon. When the AA-treated mice were irradiated on day 0, the area of damage tended to be more widespread than that in nonirradiated mice on day 7. This indicated that RT delayed the healing of mucosal damage. Although the BMC count did not decrease, the WBC count decreased significantly (Figure 2(b)). In contrast, when mice were irradiated 14 days prior to inducing mucosal damage with AA at day 0 (Pre-RT), we found that the damaged area was significantly decreased on day 7 (AA versus AA + Pre-RT; Figure 2(c)). This observation suggests that preirradiation favored the healing of mucosal damage. During these periods, the WBC count remained lower in the irradiated mice than in the nonirradiated mice; however, the count suggested recovery, so we chose day 14 as Pre-RT. In addition, some of the monocyte-derived cytokines and chemokines were upregulated during these periods (data not shown).

### 3.3. Irradiation Affected the Proportional Change of Monocyte Subsets

**Figure 3:**
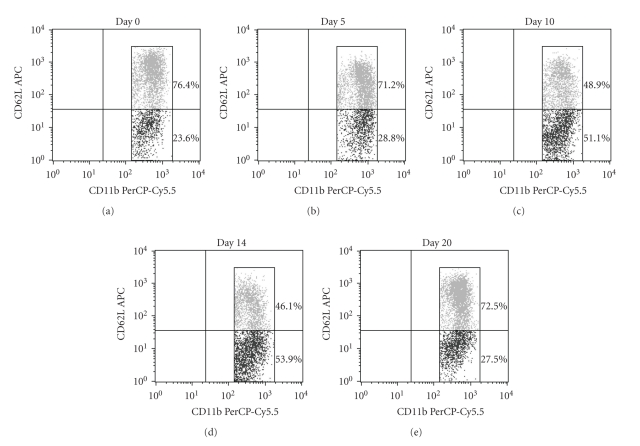
Flow cytometric analysis of peripheral blood monocyte subsets. Five mice were sacrificed at each time point after RT, and whole peripheral blood was pooled. Peripheral blood mononuclear cells (PBMC) were isolated from whole blood using gradient centrifugation method. 1 × 10^6^ PBMC were stained using two antibodies, CD11b-PerCP-Cy5.5 and CD62L-APC. CD11b**^+^**CD62L**^+^** population was designated as Subset 1, and CD11b**^+^**CD62L**^−^** population was Subset 2.

Since Pre-RT favored the healing of mucosal damage in the colon, we assumed that TBI affected not only the WBC count but also the peripheral blood monocyte subsets. As shown in Figure 3, kinetic changes in peripheral blood monocyte subsets were induced by RT. One monocyte subset, CD11b**^+^**CD62L**^+^** Subset 1, was dominant on day 0 compared with the CD11b**^+^**CD62L**^−^** Subset 2 (76.4% versus 23.6%). After RT, however, the proportion gradually changed, and the CD11b**^+^**CD62L**^−^** Subset 2 was dominant on day 10 (48.9% versus 51.1%) to day 14 (46.1% versus 53.9%), and on day 20 the proportion returned to that observed in the preirradiation state (72.5% versus 27.5%). 

In summary, the monocyte subsets changed from Subset 1 dominance to a transient increase in Subset 2 and finally returned to Subset 1 dominance. We speculate that Pre-RT favored the healing of mucosal damage shown in Figure 2(c), which may be associated with the monocyte subset changes reported herein.

### 3.4. 5-FU Treatment Delayed the Healing of Mucosal Damage

**Figure 4:**
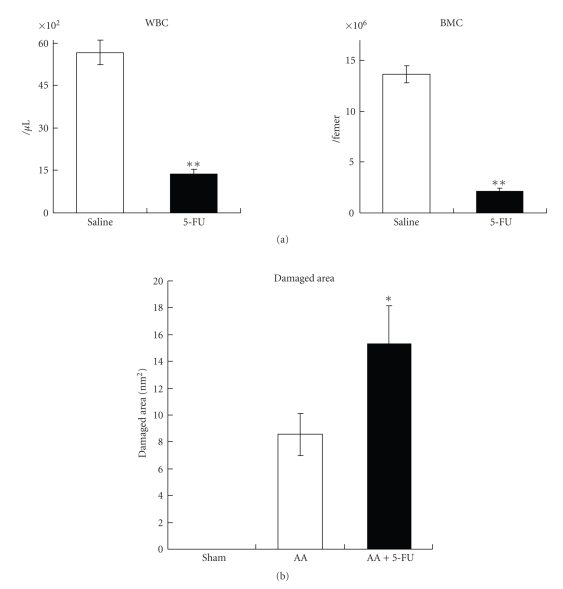
Effect of 5-FU treatment on the AA-induced mucosal damage. (a) 5-FU induced leukocytopenia and bone marrow suppression on day 7. Values represent mean ± SEM. Data were analyzed using the Welch's *t*-test. **P < .01, Saline versus 5-FU. (b) Mucosal damage was induced by AA treatment on day 0 and injected 5-FU (100 mg/kg) on the same day. 5-FU treatment exacerbated the mucosal damage and delayed mucosal healing on day 7 like the simultaneous irradiation in Figure 2(b). Values represent mean ± SEM. Data were analyzed using the Tukey method. *P < .05, AA versus AA + 5-FU.

We next examined whether the administration of an anticancer drug would induce the same phenomenon as was observed with RT. Administration of 5-FU (100 mg/kg) induced leukocytopenia and BM suppression on day 7 (Figure 4(a)). When 5-FU was administered to AA-treated mice on day 0, the damaged area increased significantly compared with that in saline-administered control mice on day 7 (Figure 4(b)); that is, 5-FU treatment delayed the healing of mucosal damage, as seen with RT. Furthermore, the delay in the healing of mucosal damage following 5-FU treatment was more severe than that with RT. We next determined the effect of BMT on the exacerbation of mucosal damage by 5-FU treatment.

### 3.5. Administration of BMCs Enhanced the Healing of Mucosal Damage

**Figure 5:**
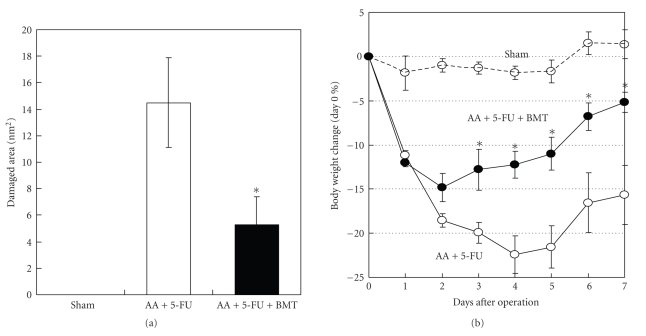
Administration of bone marrow cells (BMC) enhanced healing mucosal damage. (a) Mucosal damage was induced by the AA treatment and the additional administration of 5-FU (100 mg/kg) on day 0 (AA + 5-FU). 1 × 10^7^ BMC were injected into AA + 5-FU mice on day 1. In the Sham-operated group (control), saline was injected into colon instead of AA. Administration of BMC enhanced healing mucosal damage in AA + 5-FU mice on day 7 significantly. (b) Comparison of changes in body weight between each group. Although AA + 5-FU mice significantly reduce their body weight, BMT rescued the body weight loss from day 3. Values represent mean ± SEM. Data were analyzed using the Tukey method. *P < .05, AA + 5-FU versus AA + 5-FU + BMT.

 Since the BMC count was decreased by 5-FU treatment, we assumed that BMT would help heal the mucosal damage. Mice with AA-induced mucosal damage that were administrated 5-FU (AA + 5-FU) underwent BMT, and we found that BMT with 1 × 10^7^ BMCs showed a significantly enhanced healing of mucosal damage 6 days after BMT (>60%; Figure 5(a)). AA + 5-FU administration caused significant body weight loss compared with the sham operation (saline-treated) group, but BMT rescued the body weight loss significantly after day 3 (versus AA + 5-FU group) (Figure 5(b)). Heavy bleeding in the stool was observed after the operation, particularly in the AA + 5-FU group.

### 3.6. Labeled BMCs Migrated into the Damaged Area of the Mucosa

**Figure 6:**
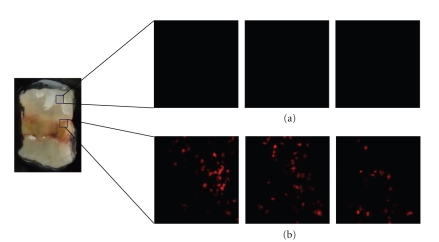
Transplanted BMC migrated into mucosal damaged area. 1 × 10^7^ BMC were labeled using Vivotag750. These cells were transplanted into AA + 5-FU model via tail vein. After 24 hours, mice were sacrificed, and the colon was observed. The labeled cells could be detected in mucosal damaged area by fluorescence microscope. Transplanted BMC accumulated significantly in mucosal damaged area (b), but not in normal area (a). Three pictures were *z*-axis high, middle, and low in one of the particular area.

We hypothesized that transplanted BMCs migrate into the damaged area of the mucosa and enhance its healing. To test this hypothesis, we injected Vivotag750-labeled BMCs into AA + 5-FU mice and determined whether the labeled cells could be detected in the damaged area using fluorescence microscopy. As shown in Figure 6, labeled BMCs (1 × 10^7^) were markedly accumulated in the damaged area compared with those in the undamaged areas of the mucosa. Thus, it is possible that the labeled BMCs were involved in the healing of the damaged mucosa.

### 3.7. F4/80^+^Gr1^+^ (Subset 1) Cells Accumulated in the Damaged Area of the Mucosa in the Early Phase of Healing

**Figure 7:**
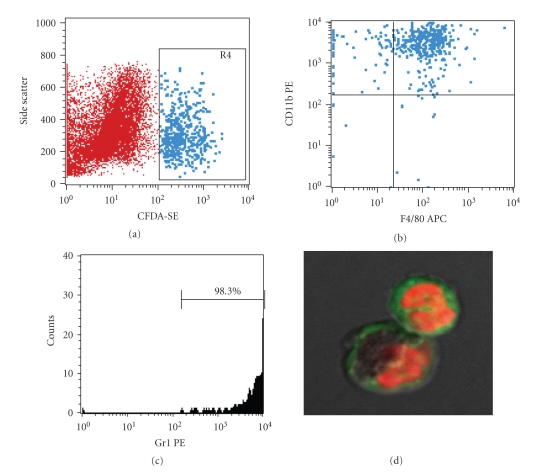
F4/80^+^CD11b^+^Gr1^+^ (Subset 1) cells accumulated at the mucosal damaged area in early phase of healing. 1 × 10^7^ BMC were labeled using CFDA-SE. These cells were injected into AA + 5-FU mice via tail vein. After 24 hours, colon was dissected and incubated in calcium and magnesium-free HBSS containing 2.5% heat-inactivated FBS and 1 mM DTT to remove mucus. After the tissues were treated with collagenase and DNase I for 60 min at 37°C, cells were washed and stained with 7-AAD, F4/80-APC, and C11b-PE or 7-AAD, F4/80-APC, and Gr1-PE, for the flow cytometric analysis. 7-AAD was used for exclusion of dead cells. (a) CFDA-SE positive cells indicated bone marrow-derived cells. (b) CFDA-SE positive population was CD11b**^+^**F4/80**^+^** monocytes. (c) F4/80 positive population was almost all Gr1**^+^**cells, indicating the F4/80**^+^**Gr1**^+^** Subset 1 monocytes. (d) The population had typical monocytes morphology.

 To further determine the characteristics of the accumulated cells, we used a CFDA-SE labeling method for flow cytometric analysis (FACS). Single cells accumulated in the damaged tissues were isolated and analyzed by FACS. The isolated cells were identified based on 1 of 3 colors. They were stained with 7-AAD, F4/80-APC, and C11b-PE or 7-AAD, F4/80-APC, and Gr1-PE. The CFDA-positive fraction originated from the transplanted BMC (Figure 7(a)), which clearly expressed CD11b and F4/80 (Figure 7(b)). However, it should be noted that the F4/80**^+^** fraction also expressed Gr1 (Figure 7(c)).

 Thus, these analyses identified the accumulated cells in the damaged area as F4/80**^+^**Gr1**^+^** (Subset 1) monocytes, which exhibited typical monocyte/macrophage morphology (Figure 7(d)).

### 3.8. Both F4/80^+^Gr1^+^ (Subset 1) and F4/80^+^Gr1^−^ (Subset 2) Cells Accumulated in the Damaged Area of the Mucosa in the Later Phase of Healing

**Figure 8:**
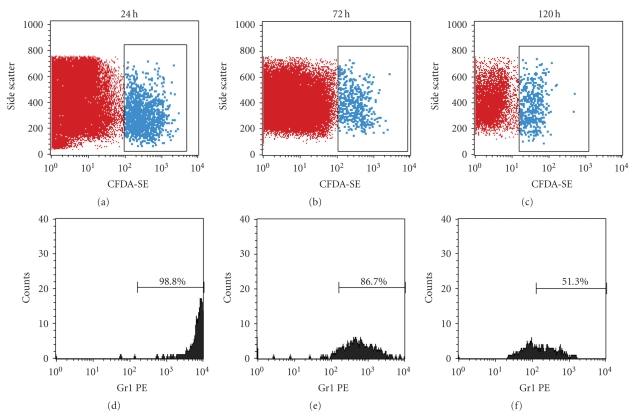
Both of F4/80^+^Gr1^+^ (Subset 1) and F4/80^+^Gr1^−^ (Subset 2) cells accumulated mucosal damaged area in late phase. Accumulated cells at the mucosal damaged area in late phase were analyzed at three time points after BMT. At 24 hours after BMT, almost all accumulating cells were F4/80**^+^**Gr1**^+^** Subset 1 monocytes (98.8%), and these Gr1**^+^** Subset 1 monocytes decreased to 86.7% after 72 hours and 51.3% after 120 hours.

Labeled BMCs (1 × 10^7^ cells) transplanted into AA + 5-FU mice were monitored for 24, 72, and 120 h by FACS. F4/80**^+^**Gr1**^+^** (Subset 1) monocytes accumulated in the damaged area of the mucosa 24 h after BMT. However, Gr1 expression was weakened 72 h after BMT (86.7%) and was further weakened at 120 h after BMT (51.3%) compared with the expression at 24 h (98.8%), (Figure 8). We therefore assumed that F4/80**^+^**Gr1**^+^** (Subset 1) monocytes migrated to the damaged area of the mucosa predominantly in the early stages of healing, while F4/80**^+^**Gr1**^−^** (Subset 2) monocytes accumulated in the area during the later phase.

## 4. Discussion

Since RT or anticancer drug administration induces not only leukocytopenia but also BM suppression, we hypothesized that these treatments might delay the healing from mucosal damage. We surprisingly found that preirradiation accelerated the healing of mucosal damage in mice that were irradiated before the colon was damaged by AA treatment. We also found that BMT significantly enhanced the healing of mucosal damage induced by AA + 5-FU treatment. These observations strongly suggested that BMCs or a BMC-derived cell population contributed to mucosal regeneration following damage. We thus determined the cell type accumulated in the mucosal lesion and investigated which monocytes or monocyte subset played a critical role in the healing of mucosal damage caused by irradiation or chemotherapy. 

 As mentioned before, mucosal damage is a common side effect of many cancer treatments, and unfortunately there is no therapy for this disorder as yet [1–3]. Intestinal and oral mucositis are significant clinical problems in patients receiving irradiation as the intestinal and oral mucosae are the most radiosensitive organs. It was difficult to establish the mucosal injury model using irradiation or anticancer drug alone. So, we combined AA with TBI or with 5-FU to mimic mucosal injury in the cancer patients. We also tried DSS (Dextran sodium sulfate) model, but it was more complicated because DSS has the anticoagulate effect by itself and far from the clinical context of cancer patient. Although the AA-induced mucosal injury model is an old type, it has widely been used for evaluating drug efficacy. 

 The mechanism of radiation- or chemotherapy-induced mucositis has been extensively documented [5–7] and has enabled testing of various treatment modalities in experimental animals [8–13]. Repair of tissue damage is an interactive process that involves soluble mediators, extracellular matrix components, resident cells, and infiltrating leukocytes [28, 29]. Initially, neutrophils accumulate at wound sites, which is followed by a large influx of macrophages and a small number of T lymphocytes. In the proliferative phase, massive angiogenesis occurs, and fibroblasts migrate into the wound, proliferate, and transform into myofibroblasts, which play a major role in the formation of granulation tissue. Recently, Ishida et al. using CXC_3_R1- (CXC chemokine receptor 1-) knockout mice demonstrated that recruitment of CXC_3_R1-positive monocytes/macrophages is critical for skin wound healing [28].

 Fluorescence resulting from illumination with an Argon 488 nm, DPSS 561 nm, or HeNe 633 nm laser is difficult to detect during *in vivo* imaging of the large intestine since mice on a normal diet exhibit autofluorescence; it is thus also difficult to image the contents of the colon and feces. Although the autofluorescence can be reduced with a purified diet, it does not completely disappear (data not shown). Therefore, we used the following 2 methods to detect the labeled transplanted cells. One was VT750 staining for fluorescence microscopy, and the other was CFDA-SE for FACS analysis. A 748 nm LD (laser diode) prevented autofluorescence due to diet, and in our analysis, cells and tissues in the colon could not be detected by FACS. We thus demonstrated that F4/80**^+^**Gr1**^+^** (Subset 1) monocytes/macrophages were recruited to the damaged area of the mucosa during the early phase of healing. Both F4/80**^+^**Gr1**^+^** (Subset 1) and F4/80**^+^**Gr1**^−^** (Subset 2) monocytes/macrophages accumulated in the damaged area in the later phase of the healing process. Ishida et al. [28] reported that both CX_3_CR1**^+^**CCR2**^−^** (Subset 1) and CX_3_CR1**^+^**CCR2**^+^** (Subset 2) monocytes/macrophages accumulated in skin wound sites in the late phase of healing and that these cells contributed to wound healing. Our results indicated that F4/80**^+^**Gr1**^+^** and F4/80**^+^**Gr1**^−^** monocytes/macrophages are sequentially recruited to damaged areas of the mucosa in 2 distinct phases. Subset 1 can be designated as immature monocytes/macrophages and Subset 2 as mature monocytes/macrophages. Since Subset 1 cells could possibly be classified as Subset 2 [18] cells given the continuum of development, some proportion of Subset 1 monocytes/macrophages may differentiate into Subset 2 monocytes/macrophages in the late phase of healing [18, 30, 31]. One study also demonstrated that monocytes have properties similar to those of pluripotent stem cells [32], so a particular population of monocytes may also differentiate into mucosal cells.

 Irradiation also induced upregulation of serum cytokines, including that of G-CSF and M-CSF (data not shown). G-CSF has been shown to have a clinically beneficial role in healing. Two randomized clinical studies showed that G-CSF was effective in reducing the incidence of oral mucositis caused by chemotherapy [33, 34] and also effectively helped reduce the severity of mucositis [35]. However, several studies on G-CSF and oral mucositis have also reported conflicting results. A randomized, controlled study using a prophylactic GM-CSF mouthwash [36, 37] and another randomized, controlled trial using a GM-CSF mouthwash as treatment for oral mucositis in hematopoietic stem cell transplantation patients showed no positive effects [38]. Although still controversial, topical administration of GM-CSF appears to have some beneficial effects on oral mucosal damage, its duration, and severity [39, 40].

 Monocytes, a complex leukocyte population that expresses a range of chemokine receptors, are generated in BM and then released into circulation [17]. Mouse monocytes are classified according to their expression of CCR2, CD62L (L-selectin), Gr1, and CX_3_CR1 [17, 18]. To functionally distinguish two mouse monocyte subsets, Geissman et al. [17] adoptively transferred GFP**^+^** monocytes from CX_3_CR1GFP/**^+^**mice to naive and immunologically challenged mice and studied GFP**^+^** monocyte cell fate and function. They found that one monocyte subset expressed CCR2, CD62L, Gr1, and only moderate amounts of CX_3_CR1, while the other subset did not express CCR2 or CD62L but expressed higher amounts of CX_3_CR1. The monocyte subset expressing CCR2**^+^**, CD62L**^+^**, Gr1**^+^**, and CX_3_CR1^low^ was designated the “inflammatory” subset (Subset 1). This subset was rapidly recruited to the sites of experimentally induced inflammation, but was short-lived after adoptive transfer, and became difficult to detect in the tissues of naive recipients [17, 19–21]. On the other hand, the CCR2**^−^**, CX_3_CR1^high^ monocyte population was found to persist longer in mice after adoptive transfer, and this population was detected in the blood, spleen, lungs, liver, and brain of recipients for several days after transfer. This population was proposed to constitute “resident” monocytes (Subset 2) that are recruited to tissues independently of inflammatory stimuli; these cells were also assumed to differentiate into other populations. Recently, increasing evidence has demonstrated that monocytes/macrophages may contribute to wound healing following vascular injury [22], spinal cord injury [23], and myocardial infarction [24]. Still other studies have provided supporting evidence that monocytes/macrophages may promote wound healing after skin injury [25–28]. The relationship between monocyte/macrophage subsets and gastrointestinal mucositis has remained unknown to date. However, in the present study, we revealed a role played by accumulating monocytes in AA-induced gastrointestinal mucositis. In summary, using an animal model, we showed that BM-derived cells, particularly monocytes/macrophages, contribute to regeneration after mucosal damage.

## 5. Conclusion

 Acetic acid- (AA-) induced mucosal damage of mouse colon was worsened by simultaneous treatment with irradiation or 5-FU. However, irradiation 14 days prior to AA treatment or BMT augmented recovery from the mucosal damage. We confirmed that F4/80**^+^**Gr1**^+^** “inflammatory” monocytes accumulated in the damaged area of the mucosa in the early phase of healing. This study suggests that monocytes/macrophages contribute to recovery and regeneration following mucosal damage.

## Abbreviations

AA: acetic acid
BMT: bone marrow transplantation
TBI: total body irradiation
RT: radiotherapy
BMCs: Bone marrow cells
PE: Phycoerythrin
PerCP: Peridinin Chlorophyll Protein-Cyanin
APC: allophycocyanin
CFDA-SE: Carboxyfluorescein diacetate-succinimidyl ester
CXCR: CXC chemokine receptor
CCR: CC chemokine receptor
WBCs: White blood cells.
